# Opioid Analgesia Following Pediatric Adenotonsillectomy: A Randomized Clinical Trial

**DOI:** 10.1002/ohn.1280

**Published:** 2025-05-21

**Authors:** Rachel L. Whelan, Jennifer L. McCoy, Leonid Mirson, Raymond C. Maguire, Noel Jabbour, Jeffrey P. Simons, Joseph E. Dohar, Dennis J. Kitsko, Amanda L. Stapleton, Allison B.J. Tobey, Cuneyt M. Alper, Amber D. Shaffer, Zachary R. Bennett, David H. Chi

**Affiliations:** ^1^ UPMC Children's Hospital of Pittsburgh, Division of Pediatric Otolaryngology Pittsburgh Pennsylvania USA; ^2^ University of Pittsburgh School of Medicine Pittsburgh Pennsylvania USA; ^3^ Present address: Office of Research and Development, Veterans Affairs Pittsburgh Healthcare System Pittsburgh Pennsylvania USA

**Keywords:** opioid prescription, pediatric opioid, postoperative opioid, postoperative pain, tonsillectomy pain

## Abstract

**Objective:**

To compare the safety and efficacy of nonopioid versus opioid pain management following adenotonsillectomy (AT) among pediatric patients.

**Study Design:**

An open‐label randomized controlled trial.

**Setting:**

Tertiary care children's hospital.

**Methods:**

Patients aged 3 to 17 years undergoing AT were eligible. Participants were randomly assigned to receive either acetaminophen and ibuprofen (nonopioid group) or acetaminophen, ibuprofen, and oxycodone (opioid group). Pain scores and prevalence of emergency department (ED) visits, hospital readmission, and posttonsillectomy hemorrhage (PTH) were compared between groups.

**Results:**

From January 2019 to March 2020, 267 patients were enrolled and randomly assigned; 144 completed a postoperative pain diary. Of the 144, 69 (48%) patients received an opioid prescription, and 75 (52%) did not. Mean pain scores before (opioid: 5.78, 95% CI: 5.29‐6.27 vs nonopioid: 5.66, 95% CI: 5.20‐6.12) and after (opioid: 2.33, 95% CI: 1.89‐2.78 vs nonopioid: 2.24, 95% CI: 1.82‐2.66) analgesics were not significantly different between opioid and nonopioid groups. Although 7/75 (9%) from the nonopioid group crossed over and requested opioids, only 43/69 (62%) randomly assigned to receive opioid prescription consumed opioids. The rate of opioid consumption increased with increasing age: 18/71 (25%) patients aged 3 to 7 years, 22/57 (39%) 8 to 12 years, and 10/16 (63%) 13 to 17 years, *P* = .015. Differences in ED visits, hospital readmissions, and PTH between opioid and nonopioid groups were not significant.

**Conclusion:**

Many children do not require opioid analgesics following AT, particularly children less than 8 years of age. Postoperative pain scores and outcomes were similar in opioid versus nonopioid groups. Opioid prescriptions should be limited or avoided altogether after pediatric AT.

**Trial Registration:**

Title: Nonopioids for analgesia after adenotonsillectomy in children; ID: NCT03618823, Clinicaltrials.gov.

In the era of the opioid epidemic sweeping the United States, surgeons across all disciplines have begun to question the optimal management of postoperative pain, with resultant opioid prescribing patterns varying tremendously. Although the undertreatment of postoperative pain is an independent predictor of complications after various surgical procedures,^1^, ^2^, ^3^ evidence suggests that all patients receiving opioid prescription following surgery remain at increased risk for opioid misuse long‐term.^4^, ^5^ This trend was specifically demonstrated among the pediatric population, wherein adolescent patients undergoing common pediatric procedures were found to be at increased risk for persistent opioid use 6 months postoperatively.^6^

Furthermore, although surgeons aim to adequately treat pain in the postoperative period,^7^, ^8^ patients often use less opioids than prescribed,^9^, ^10^, ^11^ thus leaving an excess to be potentially abused by patients or others.^12^ This is perhaps even more pronounced in the treatment of pediatric patients; Monitto et al demonstrated that a staggering 58% of opioid doses dispensed upon discharge were unused at the time of follow‐up with the majority of families improperly disposing of excess medication.^13^

With this delicate balance between adequate pain control and minimizing serious risks associated with even short‐term opioid prescription, historically, a lack of consensus exists regarding the optimal management of postoperative pain,^14^, ^15^ specifically in children^16^, ^17^, ^18^ after tonsillectomy.^19^, ^20^, ^21^, ^22^ Recently, recommendations were released regarding the development of a postoperative pain treatment plan after tonsillectomy as well as shared decision‐making by reviewing opioid risks and benefits. In line with this consensus, our study further investigates patient age groups who are and are not adequately managed postoperatively.^23^ Our objective was to compare the safety and efficacy of nonopioid versus opioid pain control following adenotonsillectomy (AT) among pediatric patients in a randomized clinical trial. We hypothesized that (1) postoperative pain would not be higher in patients assigned to the nonopioid‐only medication group compared to the nonopioid plus opioid medication group and (2) there would not be an increased rate of postoperative complications in the nonopioid versus opioid groups.

## Methods

A protocol was approved by the Institutional Review Board at the University of Pittsburgh Human Research Protection Office (STUDY19040036). An open‐label randomized controlled trial was implemented in which patients were randomly assigned to receive either acetaminophen and ibuprofen (nonopioid group) or acetaminophen, ibuprofen, and oxycodone (opioid group) at a large tertiary children's hospital. A power analysis was conducted using G*power 3.1.^24^ The effect size was based on our previously published retrospective study analyzing opioid prescription following AT.^25^ Pain scores were not collected in this prior study; instead, postoperative phone calls, follow‐up appointments, and emergency department (ED) visits were selected as indicators of postoperative recovery. Mean (standard deviation, SD) postoperative nursing phone calls, follow‐up appointments, and ED visits were 1.2 (1.2) when oxycodone was prescribed compared with 1.5 (1.3) when it was not prescribed. With *α* = .05, power = 0.80, and *δ* = 0.30, 145 patients would be required in each group. The authors aimed to enroll 300 total subjects due to a potential imbalance of completed study materials by participants in either of the groups. However, due to the COVID‐19 pandemic, enrollment was closed early.

All patients aged 3 to 17 years undergoing AT were screened through the use of electronic medical records (EMRs). Patients were excluded from study eligibility if any of the following were present: history of coagulopathy, comorbid chromosomal or craniofacial anomaly, caregivers with poor English language comprehension, pregnancy, allergy or contraindication to any study medication, active or chronic opioid prescription, plans to undergo concurrent surgery at the time of AT, or foster care with no legal documentation in EMR. Patients were also excluded if a sibling was randomly assigned to the opposite medication group or was not eligible for the study. Study participation was discussed at the preoperative appointment, and consent was obtained either at the time of office consultation or day of surgery. All eligible patients were block‐randomly assigned to have an equal distribution of opioid and nonopioid regimens among three age groups—young children (aged 3‐7), preadolescents (aged 8‐12), and adolescents (aged 13‐17). To reflect the age distribution of the patient population undergoing AT, age blocks were split between the total sample size to amount to 40% recruitment for ages 3 to 7 and 8 to 12 (n = 120 each) and 20% for ages 13 to 17 (n = 60). The random number generator in Excel was used for randomization and was performed by the senior research associate. Surgeons were instructed to use clinical judgment when prescribing opioids and verify that the child could communicate and localize pain. All 10 otolaryngologists in the department participated in recruitment. Surgeries performed at the main hospital and the ambulatory surgical center location were included. The surgical technique included both electrocautery and coblation at the discretion of the surgeon.

On the day of surgery, participating caregivers received a study folder with a welcome letter, patient pain diary, postoperative information, contact information, and directions for receiving compensation upon study completion. Families were informed of their child's randomized study group, as statewide legislation dictates that surgeons are required to review the safety and risks of opioid prescription when patients are less than 18 years of age.^25^ Oxycodone dosing was weight‐based. A dose of 0.05 mg/kg was prescribed with instructions for giving the medication every 4 hours only if acetaminophen (15 mg/kg) and ibuprofen (10 mg/kg) had both been given and the child was still demonstrating the inability to drink and/or communicated or behaved like pain was persistently undertreated. The total opioid supply was limited to 7 days. Investigators emphasized that those in the opioid group were not required to take any amount of oxycodone during the trial. Those in the nonopioid group had the right to request and receive oxycodone at any point during the trial.

**Figure 1:**
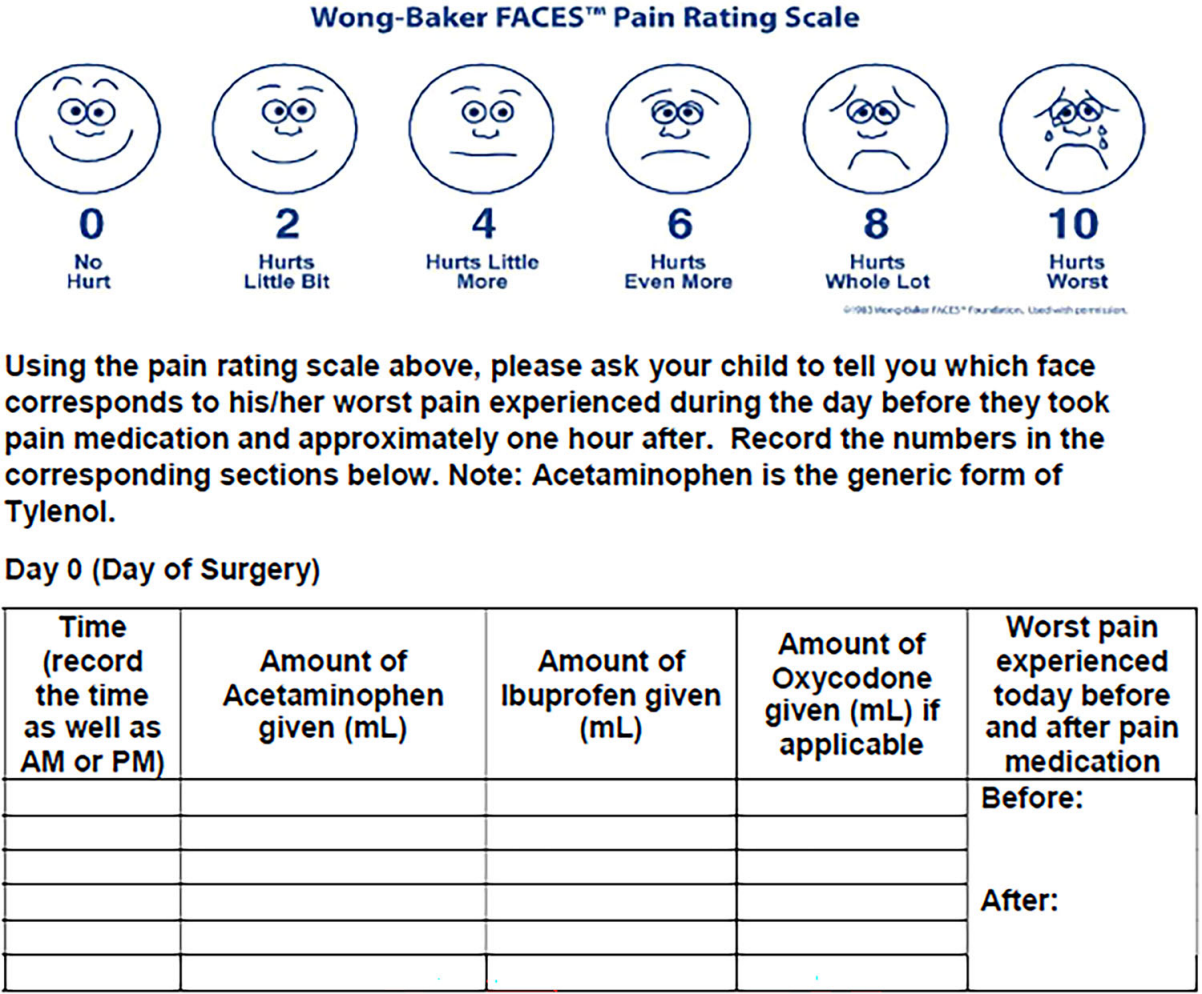
Wong‐Baker FACES Pain Rating Scale and a portion of the self‐reported pain diary.

A pain diary (Figure 1) was completed by families until postoperative day 14. Information included the amount and frequency of each pertinent medication taken, pain level using the Wong‐Baker FACES Pain Rating Scale,^26^ before and after medication, the worst pain experienced during the day, and a short outcome survey including satisfaction with pain relief. Families could return the diary electronically, by mail using a provided postmarked return envelope, or in‐person at the postoperative appointment. Compensation of $25 was provided upon return of the pain diary. Using the EMR, patient demographics, comorbidities, surgical indication, surgical technique, postoperative length of stay, complications including ED visits, hospital readmission, bleeding, need for surgical control of bleeding, and medication refills were collected. Our primary outcome measure was average pain score using the Wong‐Baker FACES Scale. Averages with 95% confidence intervals (CIs) were calculated for the first 14 days postoperatively. Averages before and after medications were calculated and analyzed separately. The secondary outcome measure was ED or urgent care visits in the first 14 postoperative days and side effects of medications: nausea, vomiting, constipation, stomachache, and difficulty breathing. Other outcome measures included the number of readmissions, the average dose and duration of each analgesic used, overall pain relief satisfaction, postoperative nursing phone calls, nighttime awakenings, group crossover, and the need for follow‐up otolaryngology appointment. There were no changes to the outcomes after the trial commenced.

SPSS v24 was used for analysis with *P* < .05 indicating significance.^27^ Two‐way mixed‐model analysis of variance was used for the primary outcome, Fisher's Exact test and logistic regression were used for the dichotomous secondary outcomes, and Kruskal‐Wallis, likelihood ratio, Mann‐Whitney *U* test, and Wilcoxon signed‐ranks test were used for additional analyses. Holm's correction was used to account for multiple comparisons.^28^ A subgroup analysis was performed between groups without drug crossover. GraphPad Prism v8 was used for figure configuration.^29^

## Results

**Figure 2:**
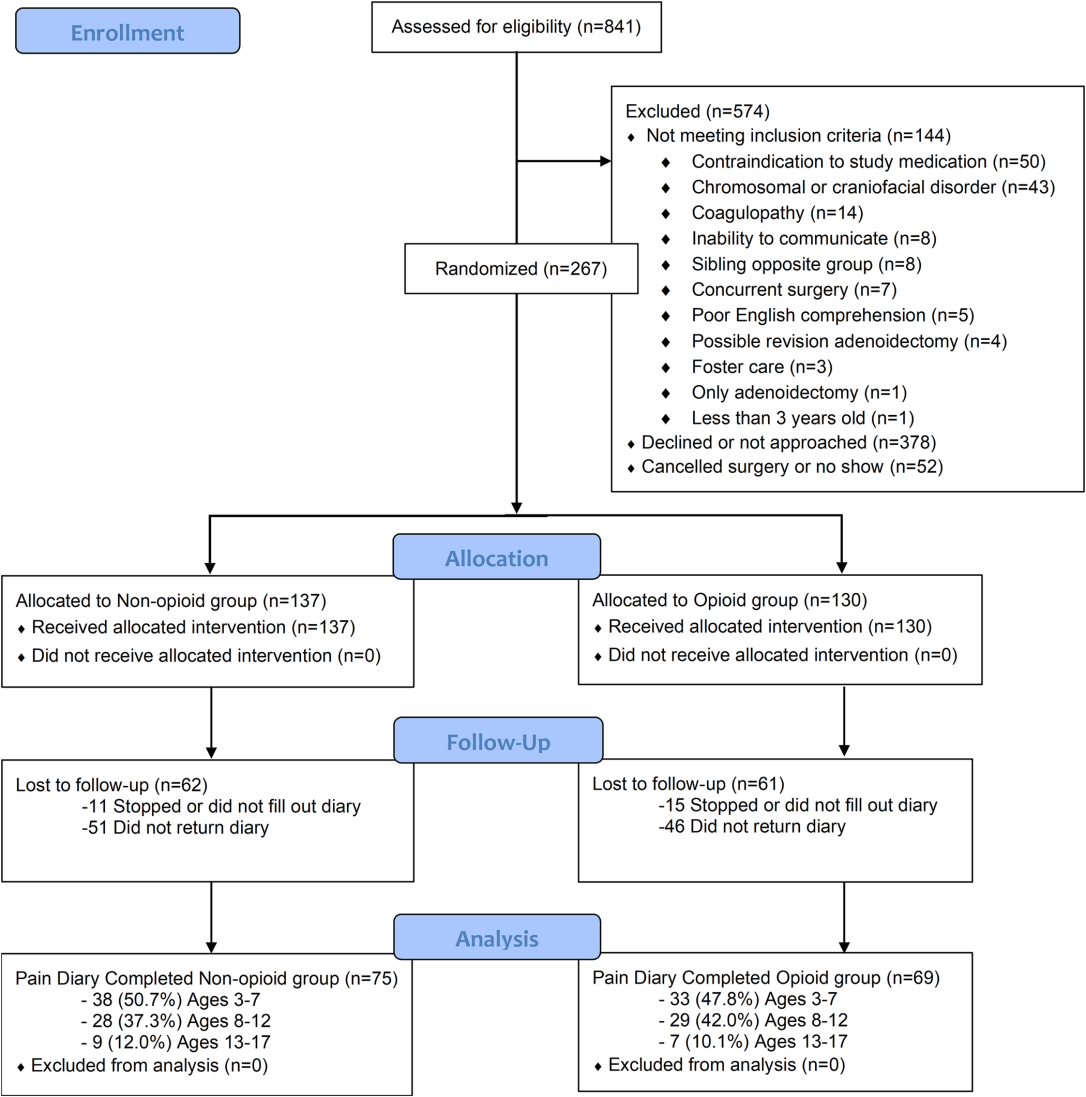
Study inclusion flow diagram (CONSORT).

Over an enrollment period spanning from January 2019 to March 2020, 267 total patients were enrolled and randomly assigned (Figure 2). The study was terminated prematurely due to the COVID‐19 pandemic. Patients who enrolled were significantly different from those who did not with respect to body mass index, race, insurance type, and American Society of Anesthesiologists status (Supplemental Table S1, available online). One hundred thirty (48.7%) patients were randomly assigned to receive an opioid prescription postoperatively, whereas 137 (51.3%) were not. Median age at time of surgery was 8 years (range 3‐17) and was not different between groups, *P* = .831. Patient demographics were not different between groups when including all participants (Table 1) and in the subset who completed pain diaries (Supplemental Table S2, available online). Indications for AT were different between opioid and nonopioid groups, but this was not significant when corrected for multiple comparisons (Table 1).

**Table 1:** Patient Demographics and Indications for Adenotonsillectomy

Demographic	All n = 267	Opioid n = 130	Nonopioid n = 137	*P* value^a^
BMI, median (range)	18.14 (12.90‐71.50)	18.11 (13.03‐71.50)	18.16 (12.90‐37.70)	.781
BMI centile, median (range)	79.22 (0.18‐99.89)	79.25 (0.18‐99.89)	77.60 (0.95‐99.82)	.826
Age at surgery, y, median (range)	8.36 (3.01‐17.72)	8.38 (3.39‐17.51)	8.28 (3.01‐17.72)	.714
Age groups, y, n (%)				.968
3‐7	120 (44.9%)	59 (45.4%)	61 (44.5%)	
8‐12	119 (44.6%)	58 (44.6%)	61 (44.5%)	
13‐17	28 (10.5%)	13 (10.0%)	15 (11.0%)	
Sex, n (%)				.807
Female	137 (51.3%)	68 (52.3%)	69 (50.4%)	
Male	130 (48.7%)	62 (47.7%)	68 (49.6%)	
Race, n (%) (n = 262)				1.000
White	237 (90.5%)	116 (90.6%)	121 (90.3%)	
Non‐white	25 (9.5%)	12 (9.4%)	13 (9.7%)	
Insurance, n (%)				.169
Public	104 (39.0%)	45 (34.6%)	59 (43.1%)	
Private	163 (61.1%)	85 (65.4%)	78 (56.9%)	
Primary reason for AT, n (%)				.050
SDB	183 (68.5%)	81 (62.3%)	102 (74.5%)	
Recurrent strep tonsillitis	75 (28.1%)	42 (32.3%)	33 (24.1%)	
Other	9 (3.4%)	7 (5.3%)	2 (1.5%)	
ASA status, n (%)				.276
1	54 (20.2%)	27 (20.8%)	27 (19.7%)	
2	201 (75.3%)	100 (76.9%)	101 (73.7%)	
3	12 (4.5%)	3 (2.3%)	9 (6.6%)	

Abbreviations: ASA, American Society of Anesthesiologists; AT, adenotonsillectomy; BMI, body mass index; SDB, sleep disordered breathing.

^a^ Continuous data were compared between opioid and nonopioid groups with the Wilcoxon rank‐sum test. Categorical data were compared between opioid and nonopioid groups with Fisher's exact test.

### Assessment of Pain Scores

**Figure 3:**
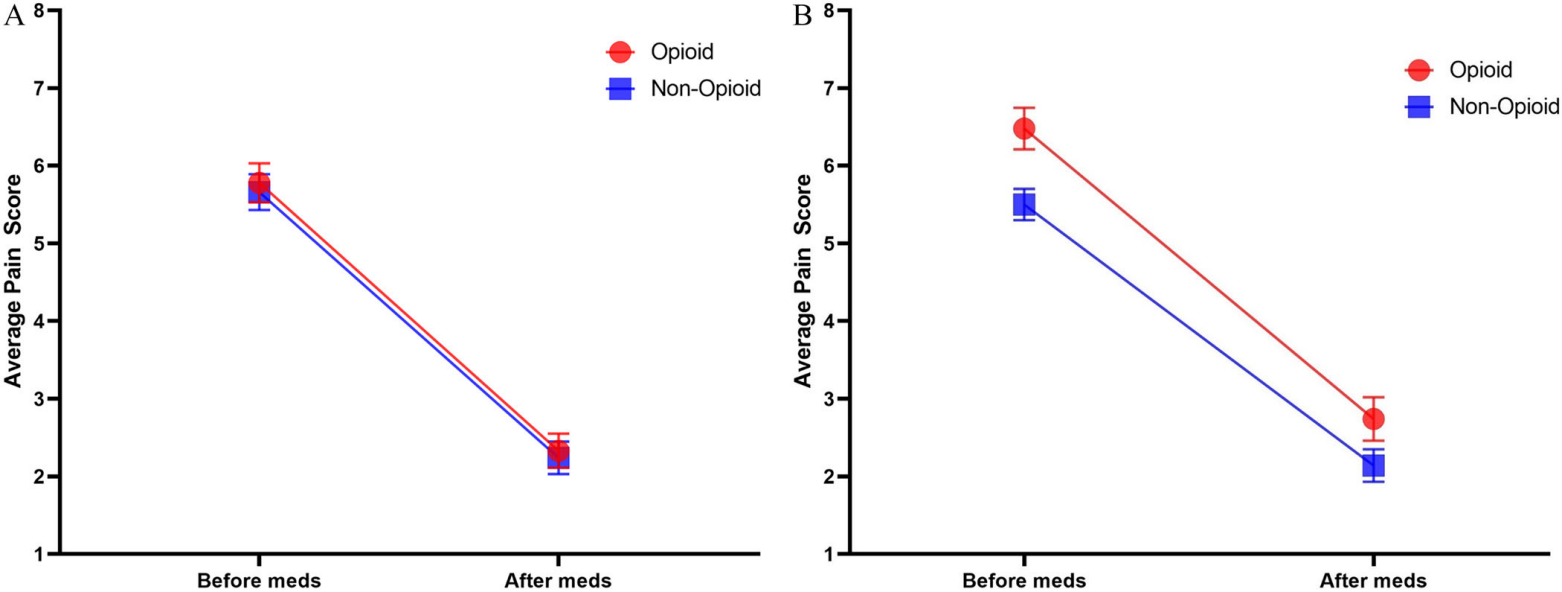
(A) Average daily pain scores between intention‐to‐treat opioid and nonopioid groups on a scale of 0 to 10, mean (standard error of the mean). (B) Subgroup analysis of average daily pain scores between patients who did not cross over between groups on a scale of 0 to 10, mean (standard error of the mean).

Postoperative pain diaries were completed by 69/130 (53.1%) of the opioid group and 75/137 (54.7%) of the nonopioid group. Demographics did not differ between those who did and did not complete pain diaries except that private insurance was more common in those who returned pain diaries (109/144, 75.7%) compared with those who did not (54/123, 43.9%) (*P* < .001) (Supplemental Table S2, available online). Pain scores were recorded before and after each dose of medication given each day. There was a significant decrease in pain after medication administration, from mean 5.51 (95% CI: 5.12‐5.90) before to 2.18 (95% CI: 1.83‐2.53) after, *F*(1,125) = 59.02, *P* < .001, ƞp^2^ = .321. There was no difference in pain scores between opioid and nonopioid groups either before medication (opioid 5.78, 95% CI: 5.29‐6.27 vs nonopioid 5.66, 95% CI: 5.20‐6.12) or after (opioid 2.33, 95% CI: 1.89‐2.78 vs nonopioid 2.24, 95% CI: 1.82‐2.66) medications, while controlling for total duration of medication use (duration mean = 10.11 days), *P* = .912 (Figure 3A). Peak pain was observed on day 6 with an average pain score of 7.00 before meds and 2.73 after meds.

There was no difference in likelihood of agreement level on a Likert scale when asked to rate the statement “I am happy with the pain relief I received the last 14 days”, *x* ^2^(4) = .166, *P* = .133. In the opioid group, 31/63 (49.2%) patients strongly agreed and 25/63 (39.7%) agreed, whereas in the nonopioid group, 25/64 (39.1%) strongly agreed and 26/64 (40.6%) agreed. There was also no difference in responses between groups when asked “How many nights did your child awaken due to pain over the last 14 days” with response options: none, some nights (1‐4), many nights (5‐9), and most nights (10‐14), *P* = .481.

### Crossover Rate and Medication Consumption

A total of 7/75 (9.3%) patients in the nonopioid group requested opioids during the trial at mean (SD) day 3.6 (2.0); median (range) = 3 (1‐7) days. The rate of crossover increased with increasing age, *P* = .034. Of patients aged 3 to 7 years, 1/38 crossed over (2.8%), 8 to 12 years, 3/28 (10.7%), and 13 to 17, 3/9 (33.3%).

Although 7 patients from the nonopioid group requested opioids, only 43/69 (62.3%) of the patients who were randomly assigned to receive an opioid prescription consumed opioids postoperatively. The rate of opioid consumption increased with increasing age: 18/71 (25%) patients aged 3 to 7 years, 22/57 (39%) 8 to 2 years, and 10/16 (63%) 13 to 17 years, *P* = .015. In total, only 50/144 (34.7%) patients consumed opioids postoperatively.

Regarding duration of pain medication administered postoperatively, median (range) use was 10.00 (3‐14) days for all patients. There was no difference in the duration of either acetaminophen (*P* = .740) or ibuprofen (*P* = .581) consumption between groups.

### Postoperative Course

Variables related to postoperative course and complication profile are described in Table 2. There was no difference in the prevalence of ED visits, hospital readmissions, or concern for tonsillar bleed between groups. Five patients in each group returned to the operating room for control of tonsillar bleed.

**Table 2:** Postoperative Course for Intention‐to‐Treat Opioid Versus Nonopioid Groups

	All n = 267	Opioid n = 130	Nonopioid n = 137	OR (95% CI)^a^	*P* value^b^
Number of nursing telephone calls, median (range)	0 (0‐4)	0 (0‐4)	0 (0‐4)	‐	.349
At least one nurse telephone call, n (%)	77 (28.8%)	35 (26.9%)	42 (30.7%)	1.20 (0.68‐2.12)	.591
Postoperative clinic appointment,^c^ n (%)	78 (29.2%)	39 (30.0%)	39 (28.5%)	0.93 (0.53‐1.63)	.888
Minor complications, n (%)^d^	55 (20.6%)	25 (19.2%)	30 (21.9%)	1.18 (0.62‐2.24)	.699
At least one postoperative ED visit, n (%)	40 (15.0%)	17 (13.1%)	23 (16.8%)	1.34 (0.65‐2.83)	.499
Postoperative ED visit for pain, n (%)	9 (3.4%)	4 (3.1%)	5 (3.7%)	1.19 (0.25‐6.15)	1.000
Postoperative ED visit for bleed, n (%)	28 (10.5%)	12 (9.2%)	16 (11.7%)	1.30 (0.55‐3.15)	.652
Hospital readmission, n (%)	27 (10.1%)	11 (8.5%)	16 (11.7%)	1.43 (0.59‐3.56)	.505
Surgical intervention for bleeding, n (%)	10 (3.8%)	5 (3.9%)	5 (3.7%)	0.95 (0.21‐4.22)	1.000
Urgent care visit, n (%), n = 131^e^	6 (4.6%)	5 (7.7%)	1 (1.5%)	0.19 (0.02‐1.63)	.128

Abbreviations: CI, confidence interval; ED, emergency department; OR, odds ratio.

^a^ Opioid was the reference group.

^b^ Continuous data were compared between opioid and nonopioid groups with the Wilcoxon rank‐sum test. Categorical data were compared between opioid and nonopioid groups with exact logistic regression.

^c^ Within 9 weeks postoperative.

^d^ Pain, epistaxis, mucus, nausea/vomiting, and dehydration.

^e^ Self‐reported via pain diary.

After pain medication administration was completed, caregivers were asked on the pain diary “Did your child experience any of the following during his or her recovery (check all that apply): nausea, vomiting, constipation, stomachache, and difficulty breathing.” In those who responded, nausea was reported in 16/65 (24.6%) in the opioid group compared to 12/66 (18.2%) in the nonopioid group, vomiting in 9/65 (13.8%) versus 5/66 (7.6%), constipation in 11/65 (16.9%) versus 6/66 (9.1%), stomachache in 18/65 (27.7%) versus 16/66 (24.2%), and difficulty breathing in 1/65 (1.5%) versus 3/66 (4.5%). None of these side effect percentages were different between groups.

### Subanalysis of Opioid Consumers Versus Nonconsumers

Given that only 43 (62.3%) patients in the opioid group took opioid medication and 68 (90.7%) patients in the nonopioid group did not request opioids, subanalysis between these two subgroups was performed. There was no difference in mean age between groups (opioid 8.79, SD 3.76 vs nonopioid 7.85, SD 3.30) (*P* = .216) and no difference in postoperative outcome measures. Additionally, there were no differences between groups with respect to premedication and postmedication pain scores when controlling for total duration of medication use, *P* = .202 (Figure 3B).

## Discussion

In working towards evidence‐based guidelines for the management of postoperative pain,^30^ this study compares opioid versus nonopioid pain control in children after AT in a prospective, randomized controlled trial. Our data suggest that most children do not require opioid prescription postoperatively without increased subjective pain scores or postoperative complications including rates of ED visits, hospital readmission, or tonsillar bleed. To our knowledge, this is the first study to randomly assign pediatric patients to receive acetaminophen and ibuprofen versus acetaminophen, ibuprofen, and oxycodone after tonsillectomy.

Only 50 of 144 (34.7%) patients required opioids postoperatively, with just over half (62.3%) of patients assigned to the opioid group administered opioids. There was no difference in average pain scores or complication profile between medication groups postoperatively. Although this statement is justified by our intention‐to‐treat analysis and our subgroup analysis of those who did not cross over, it is important to note that although statistical significance was not reached, there was a trend towards an increased number of phone calls, ED visits, and readmissions in those who did not receive opioid compared to those who did, suggesting a potential increased system‐wide burden associated with foregoing an opioid prescription.

Notably, postoperative opioid use increased with increasing age, with only 25% of patients aged 3 to 7 years compared to 63% of patients aged 13 to 17 years consuming opioids after AT. This trend is consistent with existing literature and clinical practice, wherein adolescents tend to have a more prolonged recovery, higher rates of poorly controlled pain, and ED presentation for pain following AT compared with their younger counterparts.^31^, ^32^ Although it has been suggested that differences in pain scores may be due to older children being superior at distinguishing between FACES on the pain rating scale as well as parents underestimating a younger child's pain level, there does seem to be higher pain in teenage patients.^33^ In recent literature, opioid initiation in adolescents and young adults was associated with a 30% to 40% increase in substance‐related morbidity,^34^ and the correlation between opioid prescription or family use of opioids and adolescent misuse has been well‐shown.^35^ Therefore, balancing optimal pain control with minimizing risks for opioid misuse is particularly critical in this vulnerable adolescent cohort.

Although our study is strengthened by its prospective, randomized design and incorporates both subjective measures of pain and objective measures of medication administration and clinical outcomes related to the postoperative course, limitations exist. Due to the study's nature, randomization could not be blinded. Enrollment was ended prematurely due to the start of the pandemic, most significantly limiting the number of adolescents enrolled, ultimately limiting the conclusions we can draw regarding opioid requirements and postoperative course in older patients who are most likely to experience uncontrolled pain and are also most vulnerable to potential opioid misuse risks. Our power analysis utilized previous data collected from a chart review at our tertiary care hospital.^25^ Although having the strength of utilizing data from a population very similar to that enrolled in the present study, this analysis was unable to be based on the primary outcome in this trial, pain scores after discharge. A post hoc power analysis based on data from this trial indicated that to detect a difference in mean pain scores before analgesics between opioid (mean 5.78, SD 2.08) and nonopioid (mean 5.66, SD 1.99) groups with power = 0.8 and *α* = .05, n = 9036 (4518 in each group) would be required. Even if this target was met, a 0.1 difference in pain scores on a 0 to 10 scale would be of marginal clinical significance.

The subjective nature of assessing pain and determining whether opioid was required was dependent upon parental assessment. Preconceived biases surrounding the use of opioids likely impacted decisions regarding study enrollment as well as medication administration postoperatively. Our study cohort was primarily white and privately insured. In fact, enrolled participants were more likely to be white, non‐Hispanic than those who did not meet inclusion criteria and those whose surgeries were canceled. Private insurance was also less common in those who were excluded compared with enrolled participants. However, there was no significant difference in race/ethnicity and insurance status between those who were included and those who declined or were not approached for the study. Pain is more likely to be underreported and undertreated in minority populations, thus calling the generalizability of our results into question in the larger scope of non‐white children from lower socioeconomic backgrounds.

In this study, we used the Wong‐Baker FACES Pain Rating Scale, which is used as a pain self‐report measure globally.^26^ This scale is a psychometrically reliable and validated tool used in ages 3 to 18^36^ and is commonly used in clinical trials with children due to its visual analog scale.^37^, ^38^, ^39^ We do not know whether the pain scores were reported by the patients or by proxy through caregivers. Literature has shown that caregivers both underreport^40^ or overreport pain for their child,^41^ but in general were close to^42^ or matched the child's experienced pain level.^43^ Our study design required a 14‐day pain diary to be completed by families. Given this laborious task can certainly be challenging families, it is not entirely surprising that more than 60 patients in each group were unfortunately lost to follow‐up due to failure to return/complete pain dairies. Our study did not explicitly examine socioeconomic factors, but patients who returned pain diaries were more likely to have private insurance, implying a possible economic or accessibility component to successful return of diaries and limiting the generalizability of our findings.

Our study most strongly supports limiting opioid prescription in younger children following AT. Like our study findings, Kaiser Permanente Northwest also examined a young patient cohort and found no differences in postoperative outcomes including emergency or urgent care utilization before and after implementation of guidelines that limited opioid prescription in patients <7 years old.^44^ A smaller study investigated the use of morphine and acetaminophen versus ibuprofen and acetaminophen for postoperative pain after AT and demonstrated that pain control (measured only on days 1 and 5) was adequate for both regimens, but the use of morphine was associated with complications and was deemed unsafe in certain children.^45^

Based on these results and the growing evidence base regarding efficacy and importance of multimodal pain control in the postoperative setting, the authors advise limiting or avoiding routine opioid prescription following pediatric AT. For patients who do require opioids postoperatively, we recommend limiting the prescription to an approximate 5‐day total course. Families and patients must be counseled about the risks of opioid use and safe disposal of unused medication. We also find that the era of electronic prescribing for controlled medications has made the management of delayed postoperative pain much easier for patients and families, should they indeed require opioids following discharge.

As we continue to seek a balance between achieving adequate postoperative pain control and minimizing opioid prescription, short‐term and long‐term risks associated with opioid prescription and potential misuse must be kept in mind. This study demonstrates that children rate their posttonsillectomy pain similarly regardless of having opioids and that opioids may not be necessary. Continued studies are needed as we work towards the development of evidence‐based guidelines for the treatment of postoperative pain across surgical disciplines and age ranges.

## Conclusions

Our data suggest that many children do not require opioids following AT, particularly children less than 8 years of age. Postoperative pain scores and complication profile were similar in opioid versus nonopioid groups. Opioid prescriptions should be limited or avoided altogether after pediatric AT.

## Author Contributions

**Rachel L. Whelan**, conception and design of work, interpretation of data, drafting and revision of article, approval of version to be published. **Jennifer L. McCoy**, design of work; acquisition, analysis, and interpretation of data; drafting and revision of article; approval of version to be published. **Leonid Mirson**, design of work, acquisition of data, revision of article, approval of version to be published. **Raymond C. Maguire**, design of work, interpretation of data, revision of article, approval of version to be published. **Noel Jabbour**, design of work, interpretation of data, revision of article, approval of version to be published. **Jeffrey P. Simons**, design of work, interpretation of data, revision of article, approval of version to be published. **Joseph E. Dohar**, design of work, interpretation of data, revision of article, approval of version to be published. **Dennis J. Kitsko**, design of work, interpretation of data, revision of article, approval of version to be published. **Amanda L. Stapleton**, design of work, interpretation of data, revision of article, approval of version to be published. **Allison B.J. Tobey**, design of work, interpretation of data, revision of article, approval of version to be published. **Cuneyt M. Alper**, design of work, interpretation of data, revision of article, approval of version to be published. **Amber D. Shaffer**, design of work, acquisition and analysis of data; revision of article, approval of version to be published. **Zachary R. Bennett**, acquisition and interpretation of data, revision of article, approval of version to be published. **David H. Chi**, conception and design of work, interpretation of data, revision of article, approval of version to be published.

## Disclosures

### Competing interests

There are no conflicts of interest to disclose for any author.

### Funding source

This project was funded by the Children's Hospital of Pittsburgh Foundation.

## Supporting information

Tonsil pain study supplemental tables.
