# Supplementary material for: Opioid Analgesia Following Pediatric Adenotonsillectomy: A Randomized Clinical Trial
Source: Otolaryngol Head Neck Surg. 2025 May 21;173(2):392–401. doi: 10.1002/ohn.1280 (PMC12312295; doi:10.1002/ohn.1280)
Supplement: Supplementary file 1 — Tonsil pain study supplemental tables. [file OHN-173-392-s001.docx]

Supplemental Table 1. Patient demographics and indications for adenotonsillectomy for all patients assessed for eligibility

| Demographic | Included (n=267) | Excluded:  Not meeting inclusion criteria  n=144 | Excluded:  Declined or not approached  n=378 | Excluded:  Cancelled surgery or no show  n=52 | p value* |
| --- | --- | --- | --- | --- | --- |
| BMI, median (range) | 18.14 (12.90-71.50) | 19.32 (12.60-56.81) | 17.50 (12.46-47.46) | – | .018 |
| BMI Centile, median (range) | 79.22 (.18-99.89) | 85.12 (.02-99.99) | 73.07 (.06-100.00) | – | **.006** |
| Age at surgery in years, median (range) | 8.36 (3.01-17.72) | 8.15 (2.77-17.39) | 8.15 (3.02-17.69) | – | .444 |
| Sex, n (%) |  |  |  |  | .713 |
| Female | 137 (51.3%) | 78 (54.2%) | 211 (55.8%) | 27 (51.9%) |  |
| Male | 130 (48.7%) | 66 (45.8%) | 167 (44.1%) | 25 (48.1%) |  |
| Race, n (%)† |  | ‡ |  | ‡ | **.001** |
| White | 237 (90.5%) | 114 (82.0%) | 313 (86.0%) | 34 (68.0%) |  |
| Non-White | 25 (9.5%) | 25 (18.0%) | 51 (14.0%) | 16 (32.0%) |  |
| Insurance, n (%) |  | ⸹ |  | – | **.001** |
| Public | 104 (39.0%) | 81 (56.3%) | 151 (40.0%) |  |  |
| Private | 163 (61.1%) | 63 (43.8%) | 227 (60.1%) |  |  |
| Primary reason for AT, n (%) |  |  |  | – | .039 |
| SDB | 183 (68.5%) | 105 (72.9%) | 238 (63.0%) |  |  |
| Recurrent Strep Tonsillitis | 75 (28.1%) | 32 (22.2%) | 131 (34.7%) |  |  |
| Other | 9 (3.4%) | 7 (4.9%) | 9 (2.4%) |  |  |
| ASA Status, n (%) |  | ⸹ | ⸹ | – | **<.001** |
| 1 | 54 (20.2%) | 18 (12.5%) | 110 (29.1%) |  |  |
| 2 | 201 (75.3%) | 106 (73.6%) | 261 (69.1%) |  |  |
| 3 | 12 (4.5%) | 20 (13.9%) | 7 (1.9%) |  |  |

Abbreviations: AT, adenotonsillectomy; BMI, body mass index; M, mean; SD, standard deviation; SDB, sleep disordered breathing; ASA, American Society of Anesthesiologists

†262 of those included, 139 of those not meeting inclusion criteria, 364 of those who declined participation or were not approached, and 50 of those who did not complete surgery had race reported in the electronic medical record.

‡Significantly different from included participants with Fisher’s exact test after Holm’s correction^53^ for 3 comparisons.

⸹Significantly different from included participants with Fisher’s exact test after Holm’s correction for 2 comparisons.

*Continuous data compared between included, not meeting inclusion criteria, declined or not approached, and cancelled or no show with Kruskal-Wallis tests. Categorical data compared between these groups with Fisher’s exact test.

Bold indicates significant after Holm’s correction for 8 comparisons.

Supplemental Table 2. Patient demographics and indications for adenotonsillectomy for those who completed pain diary

| Demographic | All  n=144 | Opioid  n=69 | Non-Opioid  n=75 | p value* | p value† |
| --- | --- | --- | --- | --- | --- |
| BMI, median (range) | 17.65 (12.90-71.50) | 17.50 (31.10-71.50) | 17.90 (12.90-37.70) | .920 | .147 |
| BMI Centile, median (range) | 76.25 (.18-99.80) | 77.70 (.18-99.67) | 71.60 (.95-99.80) | .877 | .279 |
| Age at surgery in years, median (range) | 8.08 (3.01-17.72) | 8.24 (3.39-17.49) | 7.97 (3.01-17.72) | .803 | .231 |
| Age groups in years, n (%) |  |  |  | .831 | .199 |
| 3-7 | 71 (49.3%) | 33 (47.8%) | 38 (50.7%) |  |  |
| 8-12 | 57 (39.6%) | 29 (42.0%) | 28 (37.3%) |  |  |
| 13-17 | 16 (11.1%) | 7 (10.1%) | 9 (12.0%) |  |  |
| Sex, n (%) |  |  |  | .616 | .463 |
| Female | 77 (53.5%) | 35 (50.7%) | 42 (56.0%) |  |  |
| Male | 67 (46.5%) | 34 (49.3%) | 33 (44.0%) |  |  |
| Race, n (%) (n=142) |  |  |  | .867 | .299 |
| White | 131 (92.3%) | 63 (92.6%) | 68 (91.9%) |  |  |
| Non-White | 11 (7.7%) | 5 (7.4%) | 6 (8.1%) |  |  |
| Insurance, n (%) |  |  |  | 1.000 | **<.001** |
| Public | 35 (24.3%) | 17 (24.6%) | 18 (24.0%) |  |  |
| Private | 109 (75.7%) | 52 (75.4%) | 57 (76.0%) |  |  |
| Primary reason for AT, n (%) |  |  |  | .562 | .628 |
| SDB | 102 (70.8%) | 46 (66.7%) | 56 (74.7%) |  |  |
| Recurrent Strep Tonsillitis | 38 (26.4%) | 21 (30.4%) | 17 (22.7%) |  |  |
| Other | 4 (2.8%) | 2 (2.9%) | 2 (2.6%) |  |  |
| ASA Status, n (%) |  |  |  | .617 | .946 |
| 1 | 30 (20.8%) | 13 (18.8%) | 17 (22.7%) |  |  |
| 2 | 108 (75.0%) | 54 (78.3%) | 54 (72.0%) |  |  |
| 3 | 6 (4.2%) | 2 (2.9%) | 4 (5.3%) |  |  |

Abbreviations: AT, adenotonsillectomy; BMI, body mass index; M, mean; SD, standard deviation; SDB, sleep disordered breathing; ASA, American Society of Anesthesiologists

*Continuous data compared between opioid and non-opioid groups with Wilcoxon rank-sum. Categorical data compared between opioid and non-opioid groups with Fisher’s exact test.

†Continuous data compared between those who completed pain diaries and those who did not with Wilcoxon rank-sum. Categorical data compared between those who completed pain diaries and those who did not with Fisher’s exact test.

Bold indicates significant after Holm’s correction for 9 comparisons.
